# Hydroxysafflor Yellow A Attenuates Lipopolysaccharide-Induced Neurotoxicity and Neuroinflammation in Primary Mesencephalic Cultures

**DOI:** 10.3390/molecules23051210

**Published:** 2018-05-18

**Authors:** Tian Wang, Yu-Xin Ding, Jie He, Cheng-Jun Ma, Yue Zhao, Zhen-Hua Wang, Bing Han

**Affiliations:** 1 Key Laboratory of Molecular Pharmacology and Drug Evaluation (Yantai University), School of Pharmacy, Ministry of Education, Yantai University, Yantai 264005, China; bluewangtian@hotmail.com (T.W.); dingyuxin1024@163.com (Y.-X.D.); 2 Collaborative Innovation Center of Advanced Drug Delivery System and Biotech Drugs in Universities of Shandong, Yantai University, Yantai 264005, China; 3 Center of Mitochondria and Healthy Aging, School of Life Science, Yantai University, Yantai 264005, China; hejie1203@163.com (J.H.); Chengjun-ma@163.com (C.-J.M.); grujwegr@163.com (Y.Z.); zhenhuawang@tom.com (Z.-H.W.)

**Keywords:** Hydroxysafflor Yellow A, lipopolysaccharide, neurotoxicity, neuroinflammation

## Abstract

Lipopolysaccharide (LPS)-induced neuroinflammation triggers and accelerates the pathogenesis of Parkinson’s disease (PD). *Carthamus tinctorius* L., a traditional Chinese medicine, has been widely used for the treatment of cerebrovascular disease. Hydroxysafflor Yellow A (HSYA) is an active component of *C. tinctorius*. The purpose of this study was to investigate whether HSYA could attenuate LPS-induced neurotoxicity and neuroinflammation in primary mesencephalic cultures. Cell viability was measured by MTT and LDH assays. The number of tyrosine hydroxylase (TH) positive neuron was observed by immunohistochemistry. NF-κB p65 and iNOS expressions were evaluated with western blotting method. Pro-inflammatory cytokines including IL-1β and TNF-α were determined by ELISA kits. Nitric oxide (NO) content in the culture medium was assayed. The results showed that HSYA treatment significantly attenuated the LPS-induced dopaminergic neurons damage. HSYA partially inhibited the expressions of NF-κB p65 and iNOS. Furthermore, HSYA decreased the content of IL-1β, TNF-α and NO in the supernatants. Taken together, these results suggest that HSYA exerts protective effects on LPS-induced neurotoxicity in dopaminergic neurons and the mechanisms may be associated with the inhibition of inflammatory response.

## 1. Introduction

Parkinson’s disease (PD) is the second most common neurodegenerative disease among the aging population. It is characterized by slow and progressive depletion of striatal dopamine as a result of the degeneration of dopaminergic neurons in the substantia nigra. Up to date, the exact mechanisms that induce the degeneration of dopaminergic neurons are poorly understood. Accumulating evidences from cellular and animal models of PD indicate that neuroinflammation plays a pivotal role in the initiation and progression of dopaminergic neurons loss [1]. Study demonstrates that chronic inflammation in the substantia nigra makes dopaminergic neurons vulnerable to degeneration [2]. Inflammation and immune responses are the determinant factors in PD progression [3]. And inflammation is responsible for the pathogenic processes in the disease onset of both familial and sporadic PD [4]. It is also reported that there are activated microglia in the substantia nigra and the putamen of PD patients [5]. The parallel changes in microglial activation and corresponding dopaminergic terminal loss in the nigrostriatal pathway of PD patients supports that neuroinflammation contributes to the degeneration process of the disease [6]. Another study suggests that different genetic mutations in genes participate directly in the progression of PD by stimulating inflammatory responses via microglia and astrocyte activation [7].

Traditional Chinese medicine (TCM) has been used for centuries to treat the tremor of head and hands which is similar to the symptom of PD. In recent times, TCM is still popular in the treatment of PD in Asian countries such as China and Korea [8]. *Carthamus tinctorius* L., a traditional Chinese medicine, has been widely used for the treatment of cerebrovascular disease. Hydroxysafflor Yellow A (HSYA) is an active component of *C. tinctorius*. Previous results in our laboratory showed that HSYA alleviated the neurotoxicity of 1-Methyl-4-phenyl-1, 2, 3, 6-tetrahydropyridine by inhibiting oxidative stress [9]. Recent study showed that HSYA inhibited LPS-induced NLRP3 inflammasome activation in mouse RAW264.7 macrophages [10]. Song and colleagues reported that HSYA suppressed the expression of TLR-4, IL-1β, TNF-α and IL-6 at the mRNA and protein levels and decreased NF-κB p65 nuclear translocation in human alveolar epithelial A549 cells [11]. HSYA suppressed the TLR4 expression in the activated microglia, resulting in a less neuronal damage at the early stage of LPS stimulation [12]. This study aimed to investigate whether HSYA could attenuate LPS-induced neurotoxicity and neuroinflammation in primary mesencephalic cultures.

## 2. Results

### 2.1. HSYA Protected Dopaminergic Neurons against LPS-Induced Neurotoxicity

**Figure 1:**
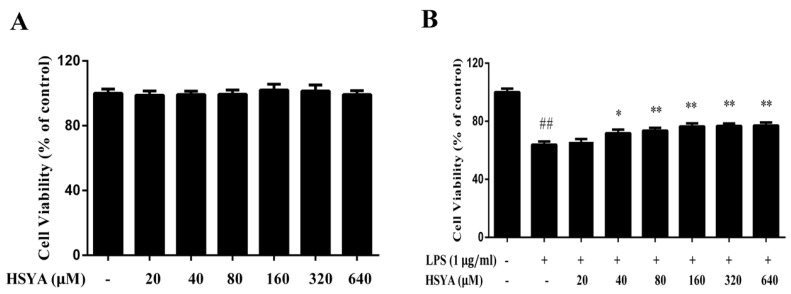
Hydroxysafflor Yellow A (HSYA) protected dopaminergic neurons against Lipopolysaccharide (LPS)-induced toxicity. (**A**) The effect of HSYA on cell viability. (**B**) The effect of HSYA on cell viability in LPS-challenged primary mesencephalic cultures. Data were expressed as the Mean ± SD of three experiments with four wells per experiment. Statistical significance was performed with one-way analysis of variance (ANOVA) followed by Tukey’s test. ## *p* < 0.01 compared with the control group. * *p* < 0.05, ** *p* < 0.01 compared with the LPS group.

Firstly, MTT assay was performed to evaluate the safe concentration of HSYA treatment on primary mesencephalic cultures. The results showed that there was no a significant difference on cell viability between the control group (100.0 ± 9.2%) and HSYA with different doses at 20 μM (98.8 ± 9.1%), 40 μM (99.2 ± 7.2%), 80 μM (99.4 ± 8.9%), 160 μM (102.0 ± 12.5%), 320 μM (101.5 ± 12.8%), 640 μM (99.3 ± 89.4%), (Figure 1A). These data indicated that the concentrations of HSYA (from 20 to 640 μM) were safety to the primary mesencephalic cultures. To investigate whether HSYA protects dopaminergic neurons against LPS-induced neurotoxicity, mesencephalic cultures were pretreated with different doses of HSYA followed by LPS challenge. Cell viability was measured by MTT at 24 h after LPS exposure. As shown in Figure 1B, decreased cell viability was observed in the LPS group (63.8 ± 7.8%, *p* < 0.01 versus control group). HSYA significantly increased the cell viability except at the dosage of 20 μM. This finding suggests that HSYA have the property of protecting dopaminergic neurons against LPS-induced neurotoxicity. To further verify the protective effect of HSYA, we used the doses of 40 and 160 μM in the following studies.

### 2.2. Effect of HSYA on LDH Release in LPS-Challenged Primary Mesencephalic Cultures

**Figure 2:**
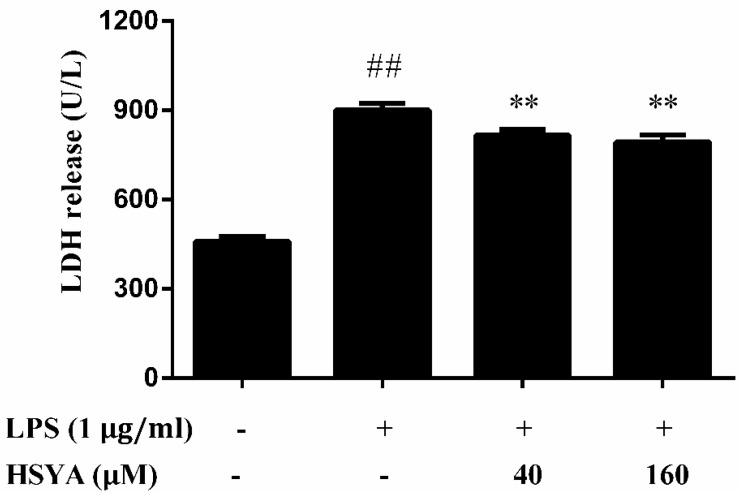
Effect of HSYA on Lactate Dehydrogenase (LDH) release in LPS-challenged primary mesencephalic cultures. Data were expressed as the Mean ± SD of three experiments with four wells per experiment. Statistical significance was performed with one-way analysis of variance (ANOVA) followed by Tukey’s test. ## *p* < 0.01 compared with the control group. ** *p* < 0.01 compared with the LPS group.

LPS exposure caused an elevated LDH release in the supernatant by 43.3% above that of control (*p* < 0.01). Pretreatment with HSYA at concentration of 40 and 160 μM partially inhibited the increase of LDH release (80.3 ± 7.3 and 75.3 ± 8.9, respectively) induced by LPS exposure (*p* < 0.01, Figure 2).

### 2.3. Effect of HSYA on TH Positive Cells in LPS-Challenged Primary Mesencephalic Cultures

**Figure 3:**
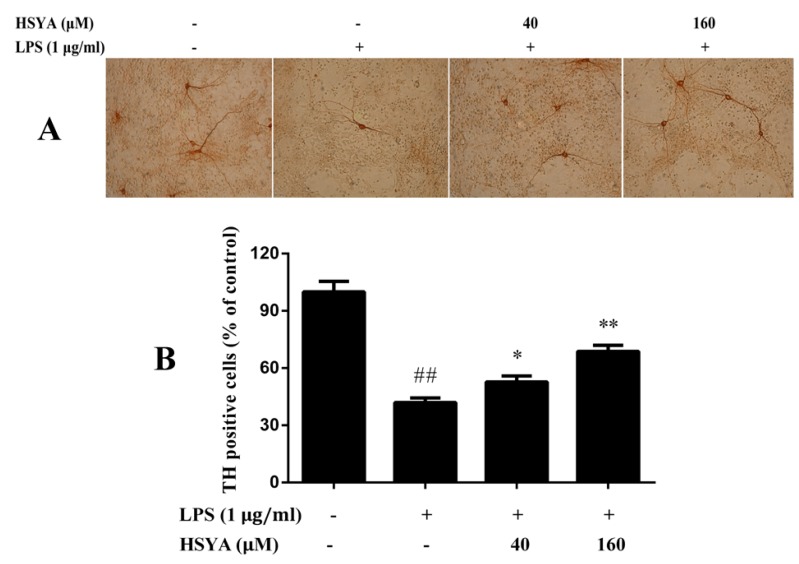
Effect of HSYA on tyrosine hydroxylase (TH) positive cells in LPS-challenged primary mesencephalic cultures. (**A**) Representative photographs of the TH positive cells in immunohistochemical staining (×100); (**B**) Bar graphs of quantitative analysis of the TH positive cells. Data were expressed as the Mean ± SD of three experiments with four wells per experiment. Statistical significance was performed with one-way analysis of variance (ANOVA) followed by Tukey’s test. ## *p* < 0.01 compared with the control group. * *p* < 0.05 or ** *p* < 0.01 compared with the LPS group.

Mesencephalic cultures exposed to LPS for 24 h were stained with TH immunoreactivity. As shown in the Figure 3, the results demonstrated that 58.1% TH positive cells were lost after LPS exposure. Pre-treatment with HSYA at concentration of 40 and 160 μM attenuated the LPS-induced reduction in the number of TH positive cells (*p* < 0.05 or *p* < 0.01).

### 2.4. Effect of HSYA on the Expressions of NF-κB p65 and iNOS in LPS-Challenged Primary Mesencephalic Cultures

**Figure 4:**
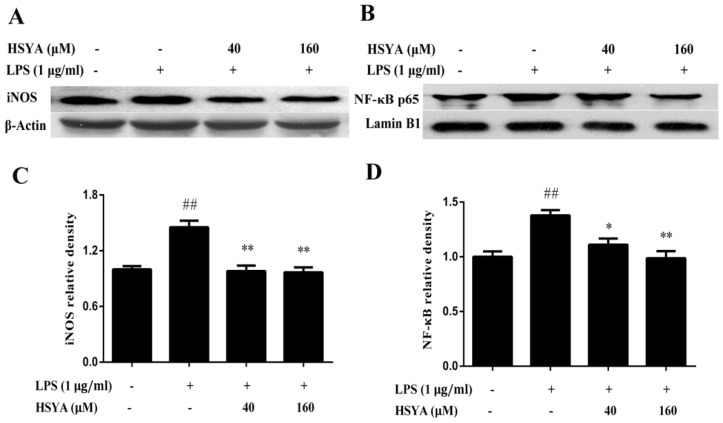
Effect of HSYA on the expressions of iNOS and NF-κB p65 in LPS-challenged primary mesencephalic cultures; (**A**,**B**) Representative photographs of the expressions of iNOS and NF-κB p65 in western blotting; (**C**,**D**) Bar graphs of quantitative analysis of iNOS and NF-κB p65, respectively. Data were expressed as the Mean ± SD of three experiments. Statistical significance was performed with one-way analysis of variance (ANOVA) followed by Tukey’s test. ## *p* < 0.01 compared with the control group. * *p* < 0.05 or ** *p* < 0.01 compared with the LPS group.

Using the western blotting analysis, we examined the effect of HSYA on the expressions of iNOS and NF-κB p65 in the mesencephalic cultures (Figure 4). Compared with the control group, the expressions of iNOS and NF-κB p65 of the LPS group significantly increased (*p* < 0.01). However, treatment with HSYA blocked the increased expressions of iNOS and NF-κB p65 induced by LPS exposure (*p* < 0.05 or *p* < 0.01).

### 2.5. Effect of HSYA on NO Levels in LPS-Challenged in Primary Mesencephalic Cultures

**Figure 5:**
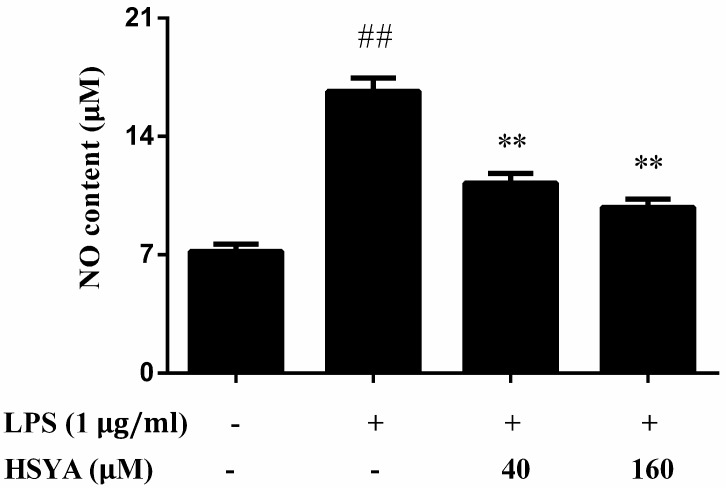
Effect of HSYA on NO levels in LPS-challenged in primary mesencephalic cultures. Data were expressed as the Mean ± SD of three experiments with four wells per experiment. Statistical significance was performed with one-way analysis of variance (ANOVA) followed by Tukey’s test. ## *p* < 0.01 compared with the control group. ** *p* < 0.01 compared with the LPS group.

The NO levels in the LPS group increased markedly as compared with that of the control group (*p* < 0.01). However, treatment with HSYA attenuated the augment of NO levels induced by LPS exposure (*p* < 0.01, Figure 5).

### 2.6. Effect of HSYA on the Contents of IL-1β and TNF-α in LPS-Challenged Primary Mesencephalic Cultures

**Figure 6:**
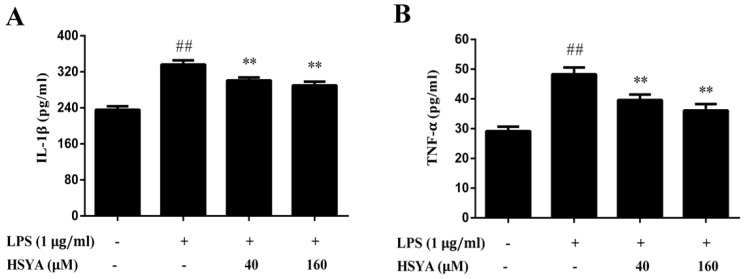
Effect of HSYA on the contents of IL-1β (**A**) and TNF-α (**B**) in LPS-challenged primary mesencephalic cultures. Data were expressed as the Mean ± SD of three experiments with four wells per experiment. Statistical significance was performed with one-way analysis of variance (ANOVA) followed by Tukey’s test. ## *p* < 0.01 compared with the control group. ** *p* < 0.01 compared with the LPS group.

At 24 h after LPS exposure, the contents of IL-1β (Figure 6A) and TNF-α (Figure 6B) significantly increased (*p* < 0.01). Compared with the LPS group, the contents of IL-1β and TNF-α in the HSYA groups significantly decreased (*p* < 0.01).

## 3. Discussion

The present study showed that HSYA treatment decreased LPS-induced neurotoxicity in dopaminergic neurons. We also observed that the protective effects of HSYA against LPS-induced neurotoxicity were associated with the decreased levels of NO, IL-1β, TNF-α and the reduction of NF-κB activation and iNOS expression. These findings suggest that HSYA exerts protective effects on LPS-induced neurotoxicity in dopaminergic neurons and the mechanisms may be associated with the inhibition of inflammatory response.

LPS is an active immunostimulant in the cell wall of Gram-negative bacteria that is responsible for initiating cytokine production. It is reported that intranigral injection of LPS results in an inflammatory reaction and then decreases both dopamine and TH levels in the striatum and the substantia nigra [13]. Other report has shown that mesencephalic cultures from substantia nigra have fourfold to eightfold more microglia than cultures from other brain regions and therefore the substantia nigra was more susceptible to LPS-induced neurotoxicity [14]. LPS induces a NF-κB-dependent transcriptional activity in brain. Upon the LPS stimulation, IκB is phosphorylated by IκB-specific kinases and IκB is degraded. Subsequently, active dimers of NF-κB will be liberated for transport into the nucleus [15]. Studies have shown that gene expression in many of the proinflammatory responses is controlled by the transcription factor NF-κB [16]. NF-κB plays a crucial role in the inflammatory response by regulating genes that encode proinflammatory cytokines, for example, IL-1β and TNF-α [17]. 

LPS also exerts its toxic effects through the inductions of iNOS and NO, which is found to play a key role in the degeneration of substantia nigra and striatum [18]. The regulation of iNOS, highly implicated in neuroinflammatory processes, takes place at the transcriptional level. Several transcription factors are associated with the activation of iNOS gene. Among them the NF-κB is the most important one. The role of NO in neuroinflammation has been determined in animal models associated with microglial activation [19]. Substantial evidence demonstrates the involvement of NO in the degeneration of dopaminergic neurons in the substantia nigra [20]. In this study, HSYA treatment significantly attenuated the LPS-induced neurotoxicity in dopaminergic neurons. The findings also showed that HSYA partially inhibited the NF-κB activity and iNOS expression. Furthermore, HSYA ameliorated the LPS-induced increase of the contents of nitric oxide, IL-1β and TNF-α. These results suggest that HSYA exerts its protective effects on LPS-induced neurotoxicity in dopaminergic neurons through the inhibition of inflammatory response.

There are existing limitations in this study. Firstly, previous result suggests that NO is one of the factors contributing to oxidative stress and oxidative damage evidenced in the PD with mortem studies and experimental models [20]. During dopamine metabolism, NO from the neurons and glial cells reacts with superoxide anion to generate reactive species like peroxynitrite which also plays a role in the injury of neuronal cells [21]. Therefore, future studies will be needed to elucidate the effects of HSYA on oxidative stress induced by LPS exposure in primary mesencephalic cultures. Secondly, it has been reported that treatment with HSYA at a dose of 180 mg/kg for 90 days results in round tubular figures and a breaking-off of the tubular epithelium in rat kidney [22]. Though the tested concentrations of HSYA (one treatment) do not affect to cell viability, the possibility of toxicity of long-term treatment with HSYA should be evaluated in future studies.

## 4. Materials and Methods

### 4.1. Materials

MTT, LPS (*E. coli* O55:B5), l-glutamic acid, sulfanilamide, *N*-(1-naphthyl) ethylenediamine dihydrochloride and paraformaldehyde were from Sigma-Aldrich (St. Louis, MO, USA). Fetal bovine serum (FBS), Dulbecco’s modified Eagle medium (DMEM, containing phenol red), horse serum, penicillin-streptomycin and DNase I were purchased from Gibco (Grand Island, NY, USA). Rabbit anti-tyrosine hydroxylase (TH) antibody were obtained from Millipore Corporation (Temecula, CA, USA). The Vectastain ABC Elite Kit (Mouse IgG) was purchased from Vector Laboratories (Burlingame, CA, USA). Rabbit anti-iNOS, anti-NF-κB, anti-Lamin B1 antibodies, HRP-conjugated goat anti-rabbit secondary antibody and Lactate Dehydrogenase (LDH) assay kit were obtained from Abcam (Cambridge, UK). ELISA kits for mouse IL-1β and TNF-α were purchased from Cusabio (Wuhan, China). Protein extraction kits, rabbit anti-β-actin antibody and ECL kit were from Beyotime Institute of Biotechnology (Shanghai, China).

### 4.2. Cell Cultures and Treatments

Pregnant C57BL6 mice were acquired from Jinan Pengyue Experimental Animal Co., Ltd. (Jinan, China). The experiments were performed according to National Institutes of Health Guidelines for the Care and Use of Laboratory Animals (publication 86-23, revised in 1986) and approved by the Institutional Animal Ethics Committee of Yantai University. Primary mesencephalic cultures of mice were established according to previous methods [23]. In brief, the ventral mesencephala were dissected from mice embryos at gestation day 14. The tissues were then mechanically and chemically dissociated into single-cell suspensions at a density of 7.5 × 10^5^ cells/mL. Cells were plated into 4-well plates pre-coated with poly-d-lysine. The cultures were incubated at 37 °C in an atmosphere of 5% CO_2_ with 100% relative humidity. The ten-day-old cultures were treated with HSYA at final concentrations (0, 20, 40, 80, 160, 320, 640 µM) with medium for 1 h before challenged with LPS (1 µg/mL). MTT assay was performed at 24 h after LPS exposure. According to the results, the concentrations of 40 and 160 µM of HSYA were chosen in the following experiments.

### 4.3. MTT Assay

Cell viability was determined by the MTT method. At 24 h after LPS exposure, MTT (0.5 μg/mL) was added into the medium and then incubated at 37 °C for 4 h. After removing the medium, 100 µL of DMSO was added to dissolve the precipitation. The absorbance at 570 nm was determined using a microplate reader (Bio-Tek, Winooski, VT, USA).

### 4.4. Immunohistochemical Staining

At 24 h after LPS exposure, cultures were rinsed with PBS (pH 7.2) and fixed in 4% paraformaldehyde for 45 min at 4 °C. Fixed cells were incubated with 0.4% Triton X-100 for 30 min at room temperature. Cultures were washed 3 times with PBS and incubated with 5% horse serum for 90 min to block non-specific binding sites. Cells were sequentially incubated with anti-TH antibody (1:1000) overnight at 4 °C. Cultures were washed with PBS 3 times. Then they were incubated with biotinylated secondary antibody for 1 h at room temperature. Total TH positive cell numbers were counted in 10 randomly selected fields at 100× *g* magnification with a microscope (IX-70, Olympus, Tokyo, Japan) by an experimenter who is blind to the treatment groups.

### 4.5. Western Blotting Analysis

The cell samples were harvested at 24 h after LPS exposure. Total proteins and nuclear proteins were extracted from cell samples using a total protein extraction kit or a nuclear extraction kit according to the manufacturer’s instructions. Add cytoplasmic extraction buffer (50 μL) to the cell pellets and then incubate on ice for 5 min. Centrifuge the tube for 5 min (14,000× *g*, 4 °C). Transfer the supernatant to a pre-chilled tube. This is the cytoplasmic protein extract. Add nuclear extraction buffer (25 μL) to the pellet and then incubate the tube on ice for 1 min. Immediately transfer the nuclear extract mixture to a pre-chilled filter cartridge with collection tube and centrifuge (14,000× *g*, 4 °C) for 30 s. Discard the filter cartridge and the flow through is the nuclear protein extract. The iNOS level was assayed in total protein extracts and the NF-κB p65 level was assayed in nuclear protein extracts. Proteins (60 µg) were conducted with 10% sodium dodecyl sulfate-polyacrylamide gel electrophoresis. Then the proteins were transferred to polyvinylidene difluoride membranes at 110 V for 1.5 h. After blocking with 5% non-fat milk for 2 h, the membranes were incubated with the primary antibodies: rabbit anti-iNOS (1:1500), rabbit anti-NF-κB p65 (1:1000), anti-Lamin B1 (1:2000), rabbit anti-β-actin (1:2000). The membranes were processed with the horseradish peroxidase-labeled secondary antibody (1:2000). Bands were visualized using the ECL kit and they were quantified using Image Quant LAS 4000 (GE Healthcare Bio-Sciences AB, Tokyo, Japan). Anti-β-actin (1:2000) served as the loading control.

### 4.6. Nitrite Assay

The NO levels in the supernatants were assayed by the Griess reaction. Briefly, 0.05% *N*-(1-naphthyl) ethylenediamine dihydrochloride, 0.5% sulfanilamide and 2.5% phosphoric acid were added to an equal volume of supernatant and incubated at room temperature for 10 min. The absorbance at 550 nm was measured with a biochemical analyzer (GENESYS 30, Thermo Scientific, Waltham, MA, USA). Fresh culture medium served as a blank.

### 4.7. Measurement of LDH Release, IL-1β and TNF-α

The culture medium was collected at 24 h after LPS exposure. LDH release, the levels of IL-1β and TNF-α in the culture medium were assayed according to the manufacturer’s instructions. Absorbance values were measured at 450 nm using the ELISA plate reader (Bio-Tek, Winooski, VT, USA).

### 4.8. Statistical Analysis

Data are expressed as the Mean ± SD of three experiments with four wells per experiment. Using SPSS 20.0 Statistical Software, statistical significance was performed with one-way analysis of variance (ANOVA) followed by Tukey’s test. A *p* value of less than 0.05 was defined statistically significant.

## 5. Conclusions

In summary, the present study suggests that HSYA exerts protective effects on LPS-induced neurotoxicity in dopaminergic neurons and the mechanisms may be associated with the inhibition of inflammatory response, at least in part.
